# The relationship between ovarian hormones and mast cell distribution in the ovaries of dromedary camel (*Camelus dromedaries*) during the follicular wave

**DOI:** 10.14202/vetworld.2023.309-316

**Published:** 2023-02-17

**Authors:** Ragab H. Mohamed, Nasra A. Yousef, Mahmoud Awad, Rasha S. Mohamed, Fatma Ali, Hassan A. Hussein, Axel Wehrend

**Affiliations:** 1 Department of Theriogenology, Faculty of Veterinary Medicine, Aswan University, Tingar, Egypt; 2 Department of Theriogenology, Faculty of Veterinary Medicine, South Valley University, 83523 Qena, Egypt; 3 Department of Histology, Faculty of Veterinary Medicine, South Valley University, Qena, Egypt; 4 Department of Animal Health, Animal and Poultry Production Division, Desert Research Center, Egypt; 5 Department of Physiology, Faculty of Veterinary Medicine, Aswan University, Aswan, Egypt; 6 Department of Theriogenology, Faculty of Veterinary Medicine, Assiut University, 71526 Assiut, Egypt; 7 Clinic for Obstetrics, Gynaecology and Andrology of Large and Small Animals with Veterinary Ambulance, Justus Liebig University, Giessen, Germany

**Keywords:** camel, follicular wave, immunohistochemistry, mast cell, ovarian hormones

## Abstract

**Background and Aim:**

Mast cells (MCs) play an essential role in regulating tissue homeostasis through various non-allergic immune reactions. This study aimed to describe the salient features of MCs during different phases of the estrous cycle and evaluate the relationship between ovarian hormones and the presence of MCs in camel ovaries.

**Materials and Methods:**

Genital tracts (n = 28) of healthy, non-pregnant camels were collected from a local slaughterhouse. The follicular wave stage was determined according to structures on the ovaries using an ultrasound device. Stages were classified as “growing” (n = 12, FØ = 0.3–0.8 cm), “mature” (n = 9, FØ = 0.9–2.2 cm), or “regression” phase (n = 7, FØ >2.5). Blood samples were collected at slaughter to determine serum estradiol-17β and progesterone levels using an immunoassay. Safranin-O, periodic acid/Schiff, alcian blue, or methylene blue stains were used to detect MCs.

**Results:**

Follicular numbers at the growing, mature, and regression phases were determined to be 36, 14, and 7 follicles, respectively. Mast cells were widely but sparsely distributed within the ovarian tissue (9.3 MCs in the growing phase, 10.7 in the mature phase, and 7.0 in the regression phase). Typical histological features of MCs were observed in ovarian stromal tissue. Some MCs were found in the interstitial tissue, either near the follicular wall or the interstitial gland. Mast cells were present at a higher density during the mature phase than in the growing and regression phases in the ovarian matrix. A significantly reduced presence of MCs was found in the regression phase than in both the growing and mature phases (p < 0.05). A very strong positive correlation was observed between serum estradiol-17β concentrations and MC density in the ovaries (r = 0.9; p < 0.001). In addition, a strong negative correlation (r = −0.65; p = 0.03) was observed between the presence of MCs and serum progesterone concentrations.

**Conclusion:**

These findings suggest that the follicular wave phase and the associated hormonal concentration induce changes in the number of MCs in the camel ovary.

## Introduction

Since the discovery of mast cells (MCs) by Paul Ehrlich in the 19^th^ century, much research has been conducted to understand further their role in the immune system [1, 2]. Recently, it was established that MCs are multifunctional immune cells involved in health and disease states [3–6]. Mast cells are crucial in various non-allergic immune responses, both innate [2, 7, 8] and adaptive [4, 9]. Their role is not limited to regulating physiological tissue homeostasis, but they also promote the evolution of peripheral tolerance in cancer cases by releasing multiple biologically active products [10]. It was determined that MCs are the major source of histamine in the bone marrow [2]. The MC receptor system is sensitive to tissue cytokines through synergistic or inhibitory interactions [2]. The primary sources of MCs are hematopoietic progenitors, which play a critical role in hypersensitivity diseases and multiple responses based on immunoglobulins [2, 11]. Furthermore, MCs are implicated in many other physiological and pathological reactions, such as wound healing, fibrosis, hypersensitivity, and neuroimmunological diseases [4, 5].

Mast cells express estradiol and progesterone receptors, the stimulation of which evokes MC degranulation [12]. Activated MCs secrete several compounds such as histamine, metalloproteinases, proteases, and vascular endothelial growth factor (VEGF). Importantly, histamine produced by MCs influences ovulation, embryo implantation, and myometrium contractility [13]. Histamine is known to increase capillary permeability and the potential for blood flow in the ovary [14]. Thus, the regulation of reproductive function by MCs is expected, as there is degradation of the extracellular matrix due to metalloproteinases and proteases, and VEGF coordinates neovascularization [15].

Morphological and functional properties of MCs differ according to their location within the organism [3]. Mast cells are reported to be widely distributed throughout female reproductive organs, including the uterus, oviduct, and ovaries of mice, rats, hamsters, cows, and guinea pigs [5, 16–18]. Previous investigation under a light microscope has revealed changes in the characteristics and location of MCs in uterine and ovarian tissues. It was noticed in cows (which demonstrate well-developed interstitial cortical stroma and a long estrous cycle) that MCs occupy the cortical and medullar tissue of the ovary [12, 19]. Other studies have highlighted that MCs exist only in the medulla in rats and guinea pigs (which have short estrous cycles) [17, 18]. Moreover, MCs were found in small numbers in the stromal tissue of the human endometrium [20, 21].

Several studies have indicated that the localization of MCs in female reproductive organs varies according to the stage of the estrous cycle in bitches [22], sows [23, 24], ewes [25], goats [26], cows [14, 27], and mares [28–30]. It has also been suggested that MCs play a role in embryo implantation in the uterus of some species [5, 31]. However, a scarce examination has been conducted to clarify the role and describe the distribution of ovarian MCs during follicular waves and pregnancy in camels. Therefore, the function of ovarian MCs needs to be elucidated. It has been suggested that during the preovulatory period, MCs are involved in increasing vascular permeability by releasing histamine [16].

Mast cells are considered novel mediators of reproductive processes and, to the best of our knowledge, there are no reported records on the correlation between follicular sizes, ovarian hormones, and MC distribution in the ovarian tissues of camels. This study aimed to assess the distribution of MCs in the ovaries of dromedary camels (*Camelus dromedarius*) during different phases of the follicular wave and to clarify the correlations between serum estradiol-17β and progesterone concentrations and distribution of MCs in camel ovaries during the breeding season.

## Materials and Methods

### Ethical approval

This study was conducted in accordance with national laws and methodsapproved by the Animal Welfare Committee of the competent regional authority (Approval no. 68/21.09.2022).

### Study period and location

Data collection took place from November 2020 to February 2021 in Aswan, Egypt. Breeding season lasted from November to April in Egypt. In comparison to other seasons, the ovary in the non-breeding season showed less activity, fewer follicles, and more interstitial tissue. In contrast, the camel’s ovary showed more activity and many follicles during the breeding season [32]. Because of this, we did not observe the MC distribution during the non-breeding season.

### Animals

**Figure-1:**
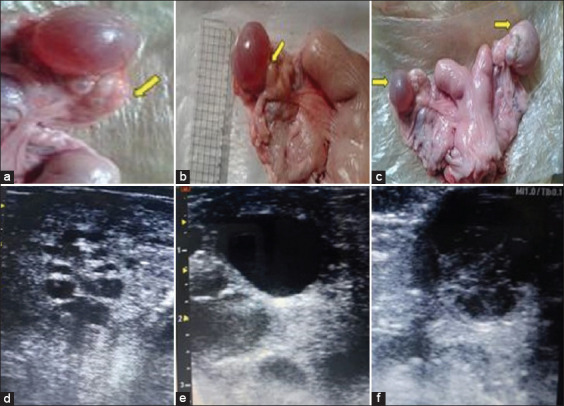
Camel ovaries containing (a, b) growing and mature follicles and (c) oversized follicles. Sonogram shows the ovary with (d) growing, (e) mature follicles, and (f) a hemorrhagic regressed follicle.

The study comprised 28 apparently healthy and non-pregnant female dromedary camels aged 8–12 years, who were slaughtered in the Darhaw slaughterhouse in Aswan. After slaughtering, the genital tracts were collected and placed in isolated containers containing ice bags (4°C) and sent to the laboratory within 1 h. The collected genital tracts were healthy and free from any genital diseases. According to ovarian findings, the samples were divided into three groups [33]. Ultrasonographic examinations were completed for all ovaries using an ultrasound device (Eickemeyer Magic 500 Digital, Germany) integrated with a linear probe (5.0–7.5 MHz). Tissue samples from the ovaries, including luteinized follicles, were classified into the following phases: Growing phase (n = 12 animals, presence of 0.3–0.8 cm follicle[s] Ø), mature phase (n = 9 animals, 0.9–2.2 cm follicle[s] Ø), and regression phase (n = 7, oversized follicle[s] >2.5 cm), as shown in Figures-1a and b [32].

### Histological examination of camel ovary

For histological preparation, ovarian tissues were trimmed into small samples and kept in formalin (10%) for 48 h. The dehydration process was achieved using an increasing concentration of ethyl alcohol (70%, 80%, 90%, and 100%) for 1.5 h for each concentration. Afterward, specimens were cleared with xylene for 60 min, and then embedded in paraffin wax. Specimen blocks were cut into histological sections of 3–5 micron thickness using a rotary microtome (Richert Leica RM 2125 Microtome, Germany). They were then stained with Safranin-O, periodic acid/Schiff, alcian blue (pH 2.5), or methylene blue stains, and then mounted with Canada balsam on cover slides for histological examinations [34]. Mast cells were identified and counted in five microscopic fields at 400× magnification.

### Blood sampling and hormonal analysis

Before the slaughter, blood samples were collected from the jugular vein. Samples were allowed to coagulate, and then centrifuged at 503× g for 10 min. Serum samples were collected and stored in 1.5 mL Eppendorf tubes at −20°C until analysis. Estradiol (Parameter™ 72, USA, Cod; KGE014) and progesterone (Oxford Biomedical Research, USA, Cod; EA74) immunoassay kits were used to detect estradiol-17β and progesterone levels in blood serum according to the manufacturer’s guidelines, as previously described [35, 36]. In estradiol-17β analysis, the intra- and inter-assay coefficients of variation were 8.9% and 12.2%, respectively. The minimum detection limit for estradiol-17β was 0.2 pg/mL. In progesterone analysis, the intra- and inter-assay coefficients of variations were 8.2% and 4.7%, respectively. The minimum detection limit for progesterone (P4) was 0.03 ng/mL.

### Immunohistochemical staining

Immunohistochemistry was performed on 5 μm sagittal cryostat sections. The ovary sections were deparaffinized in xylene and hydrated with graded alcohols. Endogenous peroxidase activity was blocked with 3% H_2_O_2_ in absolute methanol for 15 min at room temperature. Then, the sections were washed with phosphate buffer saline (PBS) at pH 7.2 and heated in citrate buffer at pH 6 in a microwave oven (700 w) for 10 min. After washing with PBS, sections were incubated with mouse primary antibody 1/100 (Mouse monoclonal tryptase, Biolegend, California, USA 369402) at 4°C for overnight. After rinsing, the sections were incubated with the secondary antibody. Diamino-benzidine was applied, and staining was carried out with hematoxylin for 1 min, followed by rinsing with tap water. The sections were then mounted using Entellan as a mounting medium.

### Statistical analysis

The results are represented by mean ± standard error (SE). One-way analysis of variance was used to compare hormonal concentrations and ovarian MC density throughout the follicular wave, and Bonferroni’s test was performed to determine significance (p < 0.05). The relationships of estradiol-17β and progesterone with MC density in the camel ovary were analyzed using Spearman’s correlation test. All statistical analyses were performed using Prism 5, GraphPad Software.

## Results

The findings of ovarian activity, monitored using ultrasound in a water bath, are presented in Table-1. Twenty-six growing follicles were observed during the growing phase (Ø = 0.3–0.8 cm), 14 mature follicles during the mature phase (Ø = 0.9–2.2 cm), and 7 oversized follicles (>2.5 cm) during the regression phase, (Figures-1a–e, respectively).

**Table-1:** Ovarian findings and estrous stage in collected samples of the dromedary camel.

Number of female animals (n=28)	Rightovary FN (Æ Mean ± SE)	Left ovary FN (Æ Mean ± SE)
Growing F (n=12)	16 (0.51 ± 0.24)	20 (0.49 ± 0.31)
Mature F (n=9)	6 (1.69 ± 0.61)	8 (1.87 ± 0.57)
Luteinizing F (n=7)	3 (3.4 ± 0.95)	4 (3.6 ± 1.03)

F=Follicle, FN=Follicular number, FW=Follicular wave, SE=Standard error

**Figure-2:**
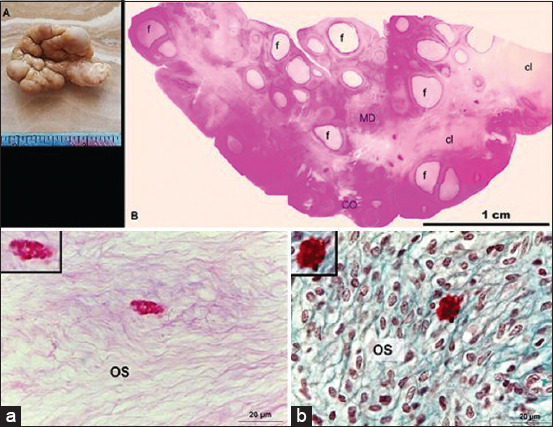
Mast cell localization in ovarian tissue of the one-humped camel. Mast cells were found in the ovarian stromal tissue (OS) either by (a) periodic acid/Schiff stain (b) or Safranin-O stain. Scale bar = 20 μm.

**Figure-3:**
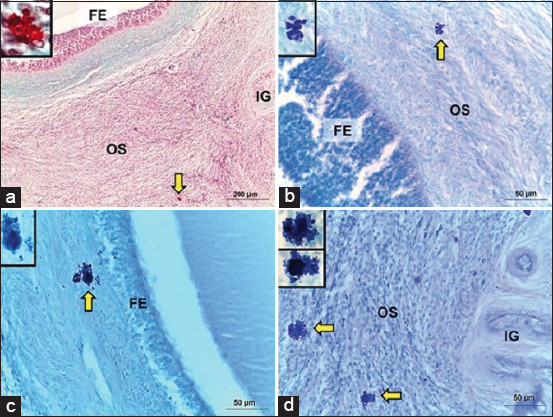
Distribution of mast cells in ovarian tissue of the one-humped camel. Ovarian tissue was stained with (a) safranin-O, (b) MB or (c and d) AB to show the distribution pattern of mast calls. (a–d) Mast cells were observed in the stromal tissue close to the outer thecal layer of the mature ovarian follicle. Mast cells were also detected within the interstitial tissue near the ovarian interstitial gland. FE=Follicular epithelium, OS=Ovarian stroma, IG=Interstitial gland. Scale bar = 200 μm (a) and 50 μm (b-d).

**Figure-4:**
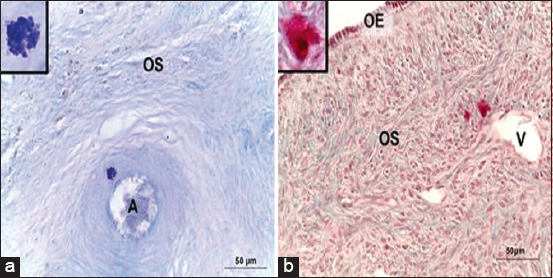
Mast cells in the ovarian tissue of a one-humped camel. Mast cells stained with (a) MB (b) or safranin-O demonstrate their localization close to blood vessels, either arteriole (a) or venule (b). OS=Ovarian stroma, OE=Ovarian epithelium, A=Arteriole, V=Venule. Scale bar = 50 μm.

**Figure-5:**
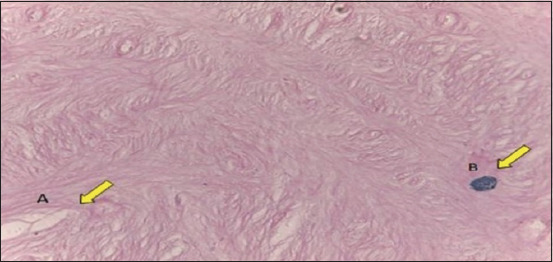
Mast cells appear in the ovarian tissue near the ovarian interstitial gland (A) interstitial gland, (B) mast cell.

**Figure-6:**
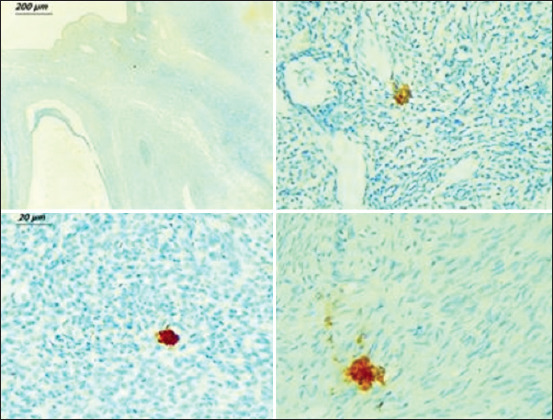
Immunohistochemistry on ovary sections confirmed mast cells in cortical regions.

A normal histological structure of camel ovarian tissue was observed in both the outer cortical and inner medullary regions. Mast cells resided in solitary numbers in the dromedary camel ovary. The ovarian cortex showed ovarian follicles in various stages of development, with no evidence of corpora lutea. However, MC density was low, as they were widely distributed in different locations within the ovarian tissue. Typical histological features of MCs with characteristic metachromatic cytoplasmic granules were observed in the ovarian stromal tissue (Figure-2). Some MCs were found in the interstitial tissue, near either the follicular wall or interstitial gland (Figure-3). Mast cells were also detected within the tunica media of some deeply situated arterioles and superficially located venules, just beneath the ovarian epithelium (Figures-4 and 5). Immunohistochemistry on ovary sections confirmed that the ovaries displayed MCs in cortical regions (Figure-6).

The average MC counts in the ovarian cortex and medulla are shown in Table-2. The number of MCs was 9.3 ± 0.3 in the growing phase, 10.7 ± 0.3 in the mature phase, and 7.0 ± 0.4 in the regression phase. There was no significant difference in the number of MCs between the growing and mature phases (p > 0.05). However, there were significant differences in the number of MCs between the regression phase and both the growing and mature phases (p < 0.05), as shown in Table-2.

**Table-2:** Mast cell density in the ovary throughout follicular wave phases (n=4 animals per phase).

Follicular wave phase	(Mean ± SE)
Growing phase	9.3^a^ ± 0.3
Mature phase	10.7^a^ ± 0.3
Regression phase	7.0^b^ ± 0.4

Means followed by different superscripts (a, b) in the same column are significantly different (p < 0.05), while values with the same superscript in the same column did not differ significantly (p > 0.05). SE=Standard error

The serum estradiol-17β and progesterone concentrations during the follicular wave phases are shown in Table-3. The highest serum estradiol-17β concentration was observed in the mature phase (149.5 ± 15.8 pg/mL). However, there were no significant differences in estradiol-17β concentration between the growing and mature phases, or between the growing and regression phases (p > 0.05). Conversely, there were significant differences in estradiol-17β concentration between the mature and regression phases (149.5 ± 15.8 and 86.8 ± 4.63, respectively, p < 0.05). In terms of progesterone concentration, no significant differences were observed between the follicular wave phases.

**Table-3:** Serum estradiol-17β and progesterone levels throughout follicular wave phases (Mean ± SE).

Follicular wave phase (number of animals)	Oestradiol-17β (pg/mL)	Progesterone (ng/mL)
Growing phase (n = 12)	114^ab^ ± 3.6	0.4 ± 0.1
Mature phase (n = 9)	149.5^a^ ± 15.8	0.5 ± 0.2
Regression phase (n = 7)	86.8^b^ ± 4.627	0.76 ± 0.1

Means followed by different superscripts (a, b) in the same column are significantly different (p < 0.05), while values with the same superscript in the same column did not significantly differ (p > 0.05). SE=Standard error

Mast cell density in the ovary was significantly influenced by estradiol-17β and progesterone concentration, as shown in Table-4. There was a very strong positive correlation between estradiol-17β and the number of MCs in the ovary (r = 0.9; p = 0.0004) and a strong negative correlation between progesterone concentration and the number of MCs in the ovary (r = −0.65; p = 0.03).

**Table-4:** Correlation between mast cells in investigated locations and peripheral hormone concentrations (estradiol-17β and progesterone) measured in blood serum.

Ovary	Oestradiol-17β	Progesterone
Correlation coefficient	0.90	−0.65
p-value	0.0004	0.03

## Discussion

The presence and quantitative distribution of MCs in the reproductive tract of many species, including bitches [22], pigs [23, 24], ewes [25], goats [26], bovines [14, 27], and mares [29, 30] have been widely discussed. Furthermore, it has been determined that there are phase-dependent fluctuations in the number of MCs during the follicular wave. However, the current study is the first to examine the spatial distribution of MCs in the ovaries of camels during the follicular wave. Although this is a valuable starting point, the functional importance of MCs in camel reproduction (male and female) is still unclear and requires further study.

In this study, MCs were not detected inside the follicles, but small amounts were located in the matrix of the medulla and the interstitial cortical stroma. Similar results suggesting that MCs tend to accumulate around blood vessels were also observed in goats [26]. The greatest number of MCs was recorded when the ovaries contained follicles of 1.8–2.0 cm (mature follicular stage), which is equivalent to the time of male tolerance in live camels (estrous period) [33]. The correlation between the number of MCs and pregnancy outcome has been previously investigated using a mouse model [37]. Mast cells seem to reach their highest level during the receptive phase (estrous) [37]. It was reported that histamine is released by MCs at the highest level immediately before the implantation phases [18, 21]. Due to its role in tissue remodeling, MC-derived histamine and matrix metalloproteinase are thought to have a beneficial effect on implantation [38, 39]. According to a study by Karaca *et al*. [40], the release of VEGF after MC degranulation promotes endothelial cell growth and angiogenesis in the uterus of pregnant rats. Before implantation, high levels of histamine decrease Th2 response while enhancing Th1, which could lead to immunologic failure [41].

However, the current study did not yield similar findings. As this study was restricted to ovarian findings of slaughtered camels who were not pregnant, further studies are required to explore the distribution of MCs in the uterus of camels during pregnancy. During human menstruation, a prevalent number of MCs were found to occupy the endometrium [42]. It was determined that MCs form a synaptic-like connection with nerve fibers in the skin and gastrointestinal tract [2, 3]. If similar connections are present in the ovary (which could not be confirmed in the present research), MCs will show membrane depolarization at these synaptic-like connections, followed by the release of cytokines and neuropeptides. These cytokines are capable of binding to appropriate nerve fiber receptors. Consequently, MCs and nerve fibers will establish a bidirectional interaction and be responsible for local neuroimmune communication. Mast cell mediators also have an effect on endothelial cells and eosinophils, which is beneficial in the recruitment of eosinophils [43, 44].

During the estrous cycle, the numbers of MCs are relatively high in the uterus, ovaries, and oviducts of cows [14] and goats [45], in the oviducts of sows [24], and in the ovaries of rats [46]. In cow ovaries, elevated MC numbers are observed in the follicular phase than in the luteal phase. Furthermore, MCs are widely distributed in the ovarian medulla. A reduced MC number is observed closer to the corpus as compared to the Graafian follicle. It can be said that MC activation regulates follicle maturation and ovulation [14, 24]. Histamine has been hypothesized to be one of the primary mediators during the ovulation process in a variety of mammals, by (i) causing the ovarian smooth muscle cells to contract more readily, (ii) promoting the production of progesterone, and (iii) having vasodilatory effects on the ovarian vasculature. In addition, serotonin and interleukin-8 produced by MCs appear to be involved in ovulation by promoting progesterone synthesis or follicular development. Proteases are vital for the breakdown of the extracellular matrix at the follicular apex during follicular rupture and can also be released in large quantities by MCs [21].

In the rat ovary, numerous MCs are detected during estrous and diestrus periods. Mast cells play a role in the vascularization of the corpus luteum [46]. An increased MC count was reported during estrous and metestrus phases. The greatest numbers of MCs were observed in the interstitial connective tissue of the cortex, ovarian medulla, and tunica albuginea [5, 47]. However, a significantly higher number of MCs was observed in pro-estrous periods in goats. The distribution of MCs was observed in the medulla and cortex interstitial connective tissue [44].

The current findings show a low number of MCs during various phases of the follicular wave relative to other species, due to the different estrous cycles in female camels. A similar observation was reported for MCs in feline ovaries studied during the estrous cycle [16]. Compared to species that are spontaneous ovulators, camels are animals with induced ovulation. Consequently, changes in their estradiol and progesterone levels during the follicular wave are not similar to those of other species. In camels, copulation or an ovulatory stimulus (induced ovulation) is needed to trigger the ovulatory process at a favorable stage of follicular development (when the expanding follicle is between 0.9 and 1.9 cm in diameter). The fate of the mature follicle can take one of two courses in the absence of mating: Atresia and disappearance in the ovarian stroma or cystic degeneration. The follicular waves have been seen to overlap. As a result, follicular development and regression cycles repeatedly occur, even in the absence of an ovulatory trigger. Furthermore, the follicular wave theory was compatible with the inverse relationship between the greatest follicle’s diameter and the total number of follicles. Estradiol concentration rises along with the dominant follicle until the follicle reaches 1.7 cm in diameter, at which point it starts to fall in dromedary camels. In the absence of ovulatory stimulation, progesterone remains at its basal level [48]. Moreover, the low number of MCs in camel ovaries may be attributed to the fixation technique effect [16]. However, further studies in camels are necessary to confirm this.

The highest concentration of estradiol was detected during the mature ovarian phase. In addition, there was a positive correlation between estrogen concentration and size of the follicle, while no variations in progesterone concentration throughout the follicular wave phases were found, supporting the results described previously [49].

The density of MCs in the ovary was higher when the serum estradiol-17β level was increased. As a result, the positive correlation of estradiol-17β with the number of MCs in the ovary was expected. The impact of ovarian hormones on the spatial distribution of MCs in reproductive organs has been investigated in many species [44]. The number and activity of MCs are positively correlated with estrogen concentrations in uterine tissue of sows and rats [18, 50] and cervical tissue of mares [30]. The activation of MCs and the subsequent induction of their degranulation could be related to the high levels of locally produced estrogen (E2) [5]. A mucosal MC analog (rat basophilic leukemia RBL2H3 cell line) was shown to be activated on incubation with E2, releasing nerve growth factor [51]. A quick initiation and gradual influx of extracellular Ca^2+^ are induced when physiological quantities of estradiol bind to a membrane estrogen receptor. This promotes the production and release of allergy mediators [52]. The number of MCs in the reproductive system of rodents has been found to be influenced by sex steroid hormones [15, 51], as are the ovary and uterus of canines [16]. In addition, the positive correlation between MC infiltration and estradiol level was confirmed in the previous study, but not with progesterone [22]. However, a correlation between the number of MCs in the canine uterine cervix and serum progesterone concentrations was reported [30]. These findings are consistent with the present study’s results, which demonstrated a positive correlation between estradiol and MC concentration in the camel ovary. On the other hand, progesterone showed a negative correlation with number of MCs in the camel ovary. These results demonstrate that although MCs play a pivotal role in the camel female genital tract, further studies are required to confirm its function.

## Conclusion

It can be concluded that the presence and quantitative distribution of MCs in the ovaries of the camels are, to a great extent, affected by follicular dynamics and hormonal changes during follicular waves. Mast cells could play an important role in camel follicular wave, but further clarification is required.

## Authors’ Contributions

HAH, AW, MA, and RHM: Designed the study. RHM, NAY, and RSM: Sample collection. MA: Histological examination, MA, NAY, FA, and RSM: Analysis and interpretation of the data. HAH and AW: Drafted the manuscript. All authors have read, reviewed, and approved the final manuscript.
